# In vitro comparative analysis of scanning accuracy of intraoral and laboratory scanners in measuring the distance between multiple implants

**DOI:** 10.1186/s40729-022-00416-4

**Published:** 2022-04-13

**Authors:** Reiji Natsubori, Shota Fukazawa, Toyokazu Chiba, Norimasa Tanabe, Hidemichi Kihara, Hisatomo Kondo

**Affiliations:** Department of Prosthodontics and Oral Implantology, School of Dentistry, Iwate Medical University, 19-1 Uchimaru, Morioka, 020-8505 Japan

**Keywords:** Intraoral scanners, Laboratory scanners, Implants, Accuracy, Distance measurement

## Abstract

**Background:**

The purpose of this study was to evaluate the accuracy of intraoral scanners by comparing the trueness and precision of several types of scanners in measuring the distance between the ball abutments on pairs of multiple implants.

**Methods:**

Seven implants were placed on a fully edentulous upper jaw model. After ball abutments were attached to the implants on the master model, the three-dimensional (3D) shape of the model was evaluated using a computer numerical control 3D coordinate-measuring machine. Subsequently, the 3D shape-related data of the model were obtained using two types of intraoral scanners (3M True Definition Scanner [TDS] and 3Shape Trios3 [TR3]) and two types of laboratory scanners (KaVo ARCTICA Auto Scan [KA] and Identica Hybrid [IH]). Using the obtained 3D shape-related data, the trueness and precision in measuring the distance between the balls within seven pairs of ball abutments were compared among the scanners using 3D analysis software.

**Results:**

Intraoral scanners produced significantly greater errors in trueness and precision than laboratory scanners in measuring the distances between the ball abutments in all the dental regions. Between the intraoral scanners, powder-requiring TDS produced significantly lower errors at inflection points than powder-free TR3.

**Conclusions:**

These results indicate that an optical impression technique using an intraoral scanner is suitable for dental implant treatment in patients with a few missing teeth.

## Background

Following the recent introduction of digital technologies into the field of dentistry, the clinical application of optical impression techniques involving the use of an intraoral scanner has become widespread. Optical impression techniques using intraoral scanners can also be used in the restoration of natural teeth with inlays, crowns, or bridges [1–3] and in dental implant treatment [4–7]. An optical impression technique requires the direct use of an intraoral scanner to record the dental structural surface shape of a patient’s oral cavity and of relevant structures within it to obtain specific information needed for administering the required treatment. Specific information is required in the following areas: treatment with tooth preparation and the status of the adjacent teeth, the presence of dental implants, opposing and adjacent dentition, and occlusion; these data are then used to construct a virtual or digitally produced three-dimensional (3D) model of the patient’s jaw. Direct intraoral scanning technology was designed to supplement or replace the physical production of models from alginate hydrocolloid or silicone elastomeric impression materials. Furthermore, intraoral scanners help to reduce uncomfortable symptoms in patients with a strong gag reflex, difficult bony prominences or protuberances with undercuts, or instances of limited or restricted mouth opening. The combination of an intraoral scanner and a computer-aided design/computer-aided manufacturing (CAD/CAM) system is expected to simplify dental restoration fabrication procedures and to reduce laboratory delivery times during patient treatment [4]. Intraoral scanners have been improved since their introduction in the 1980s. Several studies that evaluated the accuracy of impression techniques involving intraoral scanners have reported that their accuracy was comparable or superior to that of conventional impression techniques using silicone elastomeric impression materials [8–16].

Conventional dental impression techniques, requiring the fabrication of a gypsum cast, generally include errors due to the distortion of the impression material or expansion of gypsum, waxes, and investment materials. By contrast, an optical impression technique using an intraoral scanner is expected to eliminate the effect of the changes in the size of materials while taking impressions [11]. Many studies have compared the accuracy of optical impression techniques by superimposing data using a best-fit algorithm [17–23]. A best-fit algorithm is unsuitable for the measurement of an error in a particular region, because this method does not allow for arbitrary measurements, which makes the precise assessment required in dentistry difficult.

It is expected that an optical impression technique using an intraoral scanner could be used in dental implant treatment. However, much remains unknown regarding its precision. Therefore, this technique may only be suitable for single-implant restorations [24]. Thus, the application of an optical impression in the case of multiple implants has not been recommended from the viewpoint of accuracy.

Recently, several studies have been published on natural tooth-supported prostheses fabricated from digital data obtained through intraoral scanners [25–27]. However, a few studies reported the relationship between the ratio of increase in error of intraoral scanners and the scanning distance of dental arch. Furthermore, some scanners always require the use of scan powder, while others do not. Scan powder is used to enhance the scanner’s recognition ability and to shorten the scan time by reducing the reflection on the tooth surface using various materials. Furthermore, it has been previously reported that scan powder can improve scanning accuracy.

The purpose of this study was to assess the accuracy of optical impressions in relation to an increase in the scanned area. The null hypothesis of the study was that no significant differences in accuracy would be found between short-distance scanning and long-distance scanning using intraoral scanners.

## Methods

### Fabrication of the master model

**Fig. 1:**
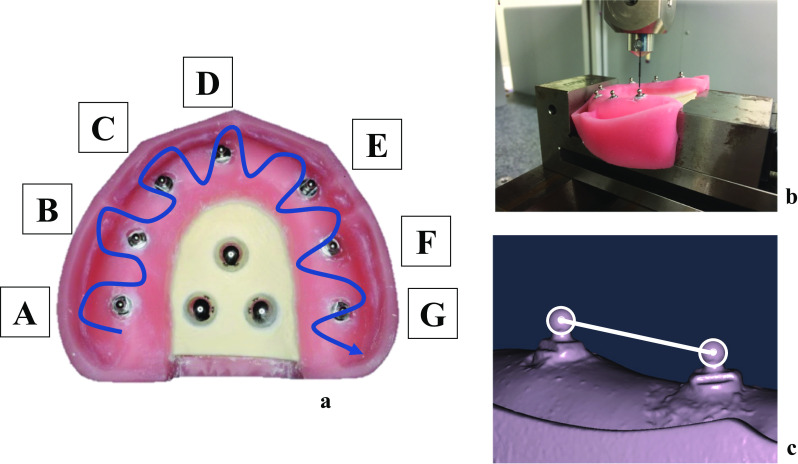
**a** Master model (direction of measurements). **b** Measurement of master model using CNC 3D coordinate-measuring machine. **c** Analysis using software

An edentulous upper jaw model (D9-EP. 29-U, Nissin, Japan) with a two-layered bony structure comprised of cortical bone and cancellous bone was employed. To create a reference plane for measuring the distance between implants, three Japan Industrial Standard (JIS) chrome steel balls (Steel Balls [Precision Balls], SUJ2, 8.5 mm, G28, Tsubaki Nakashima Co., Ltd., Japan) were fixed onto the model’s palate with an autopolymerizing resin (Provista A2, Sun Medical Co., Ltd., Japan). Cavities for implant placement were created on the jaw model. Subsequently, seven implants with an external hexagonal structure (Brånemark System MK III Groovy, regular platform, 4.0 × 10.0 mm, Nobel Biocare, Sweden) were placed in regions of the model corresponding to the upper right second molar, upper right second premolar, upper right canine, upper right central incisor, upper left canine, upper left second premolar, and upper left second molar. This model was used as a master model in subsequent experiments (Fig. 1a).

**Fig. 2:**
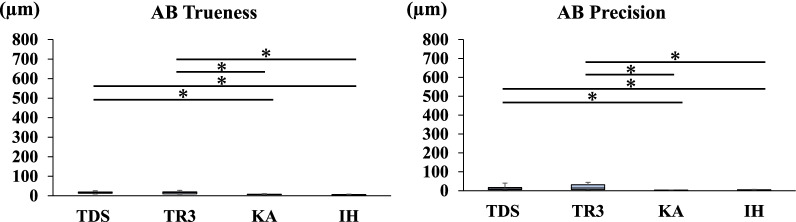
Trueness and precision in measuring the distance between A and B

**Fig. 3:**
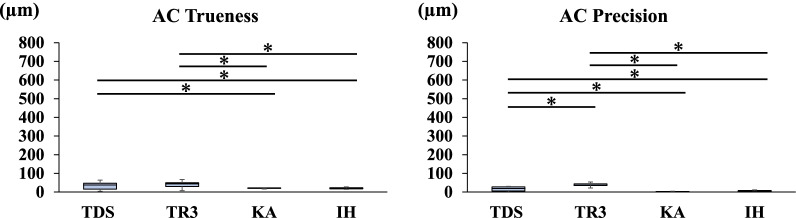
Trueness and precision in measuring the distance between A and C

**Fig. 4:**
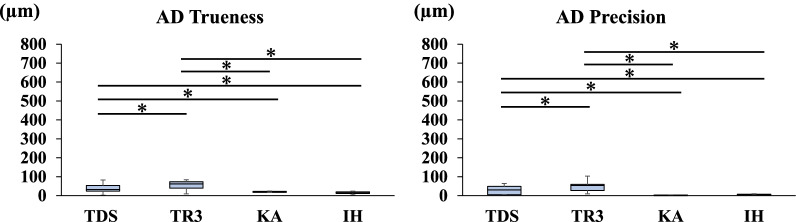
Trueness and precision in measuring the distance between A and D

**Fig. 5:**
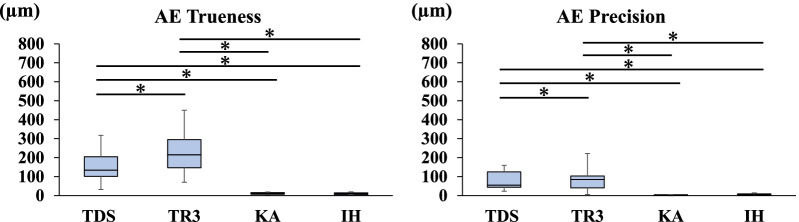
Trueness and precision in measuring the distance between A and E

**Fig. 6:**
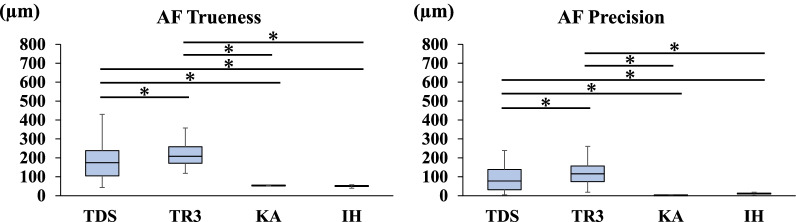
Trueness and precision in measuring the distance between A and F

**Fig. 7:**
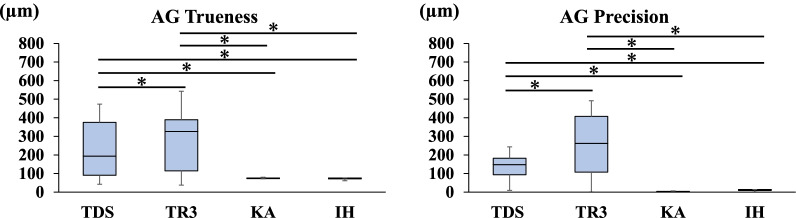
Trueness and precision in measuring the distance between A and G

A 5 × 5 mm titanium ball abutment (Brånemark System, regular platform, height 5 mm, Nobel Biocare, Sweden) was connected to the upper part of each implant placed in the master model using a prosthetic torque wrench (Nobel Biocare, Sweden) and driver (Machines Driver, Nobel Biocare, Sweden) and was tightened with a force of 15 Ncm., Nobel Biocare, Sweden) was connected to the upper part of each implant placed in the master model using a prosthetic torque wrench (Nobel Biocare, Sweden) and driver (Machines Driver, Nobel Biocare, Sweden) and was tightened with a force of 15 Ncm. This research was performed at 20 ± 0.5 °C and 50 ± 10% humidity, because the temperature should affect the accuracy of the model.

### Computer numerical control (CNC) 3D coordinate-measuring machine

The reference distances between the ball abutments in each pair of ball abutments on the master model were measured with a CNC 3D coordinate-measuring machine (UPMC 550-CARAT, Carl Zeiss, Germany). This CNC 3D coordinate-measuring machine can measure lengths with high precision. This machine conforms to the applicable JIS (JIS B7440-2), which allows a maximum error of 0.8 + L/600 µm (L = length [mm]) in the measurement of length. Before the measurement, the machine was calibrated to an error of ≤ 1 µm. The 3D position coordinates of the center of the ball on top of the ball abutment connected to each implant were measured 10 times (Fig. 1b). The mean of the 10 measurements was used as the reference value.

### Intraoral scanners

In this study, the following intraoral scanners were used: 3M True Definition Scanner (TDS, 3M, USA) and 3Shape Trios3 (TR3, 3Shape, Denmark). Before scanning with TDS, a single layer of titanium dioxide powder (Lava Powder, 3M, USA) was sprayed onto the surface of the master model, according to the manufacturer’s instructions, to minimize reflection for assessment accuracy. TR3 did not require titanium dioxide powder for assessment accuracy. The master model was secured onto a laboratory table and was scanned with intraoral scanners in a room shielded from outside light. The model was scanned by an experienced dentist 10 times per scanner according to the scan protocol of each manufacturer (Table 1). In this study, measurements were performed from A to G, as shown in Fig. 1a. After confirming that all necessary image data had been collected, the image data were converted to the standard triangulated language (STL) format.

**Table 1:** Characteristics of each scanner

	Color	Scanning principle	Powder	STL export
TDS	Blue light	Active wave front sampling	Yes	After post processing
TR3	White light	Confocal	No	Direct
KA	White light	Triangulation	Yes	Direct
IH	Blue light	Triangulation	Yes	Direct

### Laboratory scanners

The following laboratory scanners were used: KaVo ARCTICA Auto Scan (KA, KaVo Dental Excellence, Germany) and Identica Hybrid T500 (IH, Medit, Korea). Before scanning with KA and IH, a single layer of titanium dioxide powder (Lava Powder, 3M, USA) was sprayed onto the master model. The scanners were calibrated, and 10 scans were performed per scanner. After confirming that all necessary image data had been collected, the image data were converted to the STL format.

### Measurement of distances between ball abutments

The data converted to the STL format were imported into a 3D analysis software (MSURF-I, Mitutoyo, Japan). The three reference balls placed on the palate of the master model served as reference points and as a plane for the coordinate setting. The center of the ball on top of each ball abutment, which was used for the measurement of distance, was determined with a tool that visualizes the ball using analysis software. The distance between the centers of two balls was measured using position coordinates. The seven ball abutments from the upper right second molar region to the upper left second molar region were named A, B, C, D, E, F, and G, respectively. The distance between centers was determined according to the following formula (Fig. 1c):

$$\sqrt{{\left({x}_{\mathrm{A}}-{x}_{\mathrm{B}}\right)}^{2}+{\left({y}_{\mathrm{A}}-{y}_{\mathrm{B}}\right)}^{2}+{\left({z}_{\mathrm{A}}-{z}_{\mathrm{B}}\right)}^{2}}$$

where the coordinates of the center of ball abutment A were *x*_A_, *y*_A_, and *z*_A_ and those of the center of ball abutment B were *x*_B_, *y*_B_, and *z*_B_.

### Evaluation of accuracy (trueness and precision)

“Accuracy” is composed of “trueness” and “precision,” as defined by International Organization for Standardization (ISO) standard 5725-1 [28]. Trueness refers to how closely a measurement reflects the real value. High trueness indicates that the result is very close to or equivalent to the real value. Precision describes the closeness of the agreement between repetitive measurements. Higher precision implies greater consistency and predictability among the measured data. In this study, trueness was calculated as the difference between the reference distance between the ball abutments on the master model measured by the 3D coordinate-measuring machines and the distance measured by each scanner. Measurements were repeated 10 times with each scanner, and errors in the mean measured values were recorded to signify precision.

### Evaluation of error change

To evaluate the trueness and precision of the four different scanners, the error ratios of various distances were calculated according to the following formula:

$${\text{Error ratio of trueness vs. precision}}=\frac{{\text{Error value for each distance}}}{\text{Distance for each abutment}}\times 100$$

In this study, the distances AB, AC, AD, AE, AF, and AG were verified.

### Statistical analysis

A power analysis was performed using the G*Power software (G*Power, Heinrich-Heine-Universität Düsseldorf, Germany). The total sample size was calculated based on an effect size (*f*) of 0.4, an alpha error probability of 0.05, and a power of 0.8. The results of the power analysis revealed that a minimum of eight data sets per workflow was needed to perform the study. However, to increase the power, the study was performed using 10 data sets. The assumption of normality was tested using the Kolmogorov–Smirnov test. The homogeneity of variance was evaluated using Levene’s test (*P* = 0.05) in each group. Significant differences between the groups were analyzed by a one-way analysis of variance using a post hoc Bonferroni test (*P* = 0.05). Statistical analyses were performed using statistical analysis software (SPSS Statistics 24.0, IBM Japan, Japan).

## Results

Figures 2, 3, 4, 5, 6 and 7 show the trueness and precision of the scanners in measuring the distance between the ball abutments. Trueness was determined by calculating the error between the distance measured by each scanner and its corresponding reference distance. The precision of each scanner was determined by calculating the error between the mean of 10 repetitive measurements and a single measurement with the same scanner.

### Trueness and precision of AB

The mean distance between the centers of ball abutments A and B on the master model measured with the CNC 3D coordinate-measuring machine was 16.4323 mm. Regarding the trueness and precision of the scanners in measuring the distance between A and B, TDS and TR3 produced a greater error than the two laboratory scanners (*P* < 0.05) (Fig. 2).

### Trueness and precision of AC

The mean distance between the centers of ball abutments A and C on the master model measured with the CNC 3D coordinate-measuring machine was 31.7193 mm. Regarding the precision of the scanners in measuring the distance between A and C, TR3 produced a greater error than TDS. TDS and TR3 produced greater errors than the two laboratory scanners (*P* < 0.05) (Fig. 3).

### Trueness and precision of AD

The mean distance between the centers of ball abutments A and D on the master model measured with the CNC 3D coordinate-measuring machine was 44.6310 mm. Regarding the trueness of the scanners in measuring the distance between A and D, TR3 produced a greater error than TDS (*P* < 0.05). Regarding the precision of the scanners in measuring the distance between A and D, TR3 produced a greater error than TDS (*P* < 0.05). TDS and TR3 produced greater errors than the two laboratory scanners (*P* < 0.05) (Fig. 4).

### Trueness and precision of AE

The mean distance between the centers of ball abutments A and E on the master model measured with the CNC 3D coordinate-measuring machine was 53.9312 mm. Regarding the trueness of the scanners in measuring the distance between A and E, TR3 produced a greater error than TDS (*P* < 0.05). Regarding the precision of the scanners in measuring the distance between A and E, TR3 produced a greater error than TDS (*P* < 0.05). TDS and TR3 produced greater errors than the two laboratory scanners (*P* < 0.05) (Fig. 5).

### Trueness and precision of AF

The mean distance between the centers of ball abutments A and F on the master model measured with the CNC 3D coordinate-measuring machine was 52.4502 mm. Regarding the trueness of the scanners in measuring the distance between A and F, TR3 produced a greater error than TDS (*P* < 0.05). Regarding the precision of the scanners in measuring the distance between A and F, TR3 produced a greater error than TDS (*P* < 0.05). TDS and TR3 produced greater errors than the two laboratory scanners (*P* < 0.05) (Fig. 6).

### Trueness and precision of AG

The mean distance between the centers of ball abutments A and G on the master model measured with the CNC 3D coordinate-measuring machine was 52.4502 mm. Regarding the trueness of the scanners in measuring the distance between A and G, TR3 produced a greater error than TDS (*P* < 0.05). Regarding the precision of the scanners in measuring the distance between A and G, TR3 produced a greater error than TDS (*P* < 0.05). TDS and TR3 produced greater errors than the two laboratory scanners (*P* < 0.05) (Fig. 7).

**Fig. 8:**
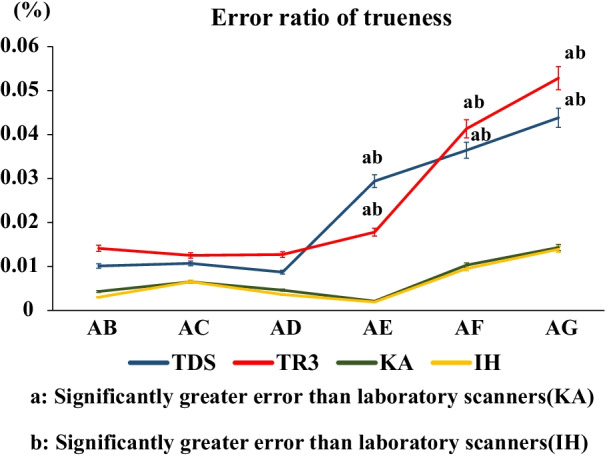
Error ratio of trueness

**Fig. 9:**
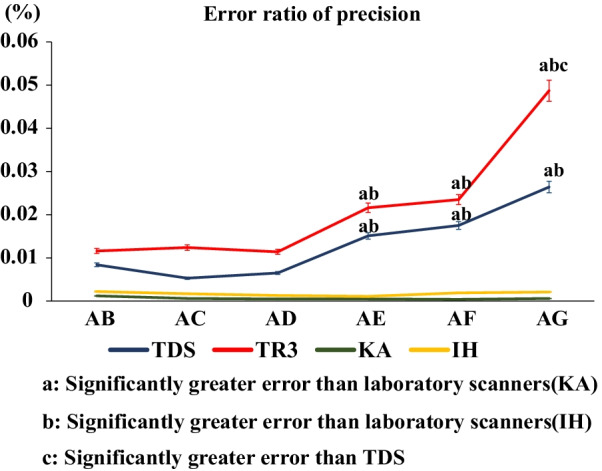
Error ratio of precision

Figures 8 and 9 show the changes in the errors in the measurements of AB, AC, AD, AE, AF, and AG. There was no significant change in the error value between AB, AC, and AD, but an increase in the error ratio was confirmed while scanning across the anterior teeth using the intraoral scanners. Regarding the error ratios of trueness and precision, TDS and TR3 produced significantly greater errors than the two laboratory scanners in measuring AE, AF, and AG (*P* < 0.05) (Figs. 8, 9). In addition, regarding the error ratio of precision, TR3 produced a significantly greater error than TDS in measuring AG (*P* < 0.05) (Fig. 9). Furthermore, AE was longer than AG, but the error variation increased between A and G. There was no significant difference between the intraoral scanners in measuring shorter distances, but there was a significant difference between them as the distance increased. However, there was no significant difference in the error ratio between the laboratory scanners (Figs. 8, 9).

## Discussion

### Clinical significance of the study

In this study, all the statistical analyses for the evaluation of accuracy led to the rejection of the null hypothesis regarding accuracy based on distance. Several studies have been published on the accuracy of optical impressions of crowns and bridges [1–3, 9, 13–16, 25–27, 29, 30]. However, in dental implant treatment, much remains unknown regarding the data obtained through an implant optical impression technique using an intraoral scanner. In particular, it has not been clarified whether this technique is accurate enough to replace the conventional method of taking impressions using silicone impression materials. This study compared the precision of intraoral scanners with that of laboratory scanners to evaluate the usefulness of an optical impression technique using an intraoral scanner.

### Research methodology

The fabrication of precise dental prostheses requires high accuracy in obtaining impressions. Various studies have reported on the trueness and precision of optical impression techniques [2, 31, 32]. Many of these studies have identified the locations of deformations by superimposing digital models using a best-fit algorithm. This technique visualizes the deformations in the entire model and shows each displacement on a color map [2, 17, 19, 29, 33].

However, the displacements identified through the abovementioned software profoundly depend on its mechanical elements, and a best-fit algorithm is unsuitable for the measurement, such as those performed in this study of an error in a particular region between the centers of the balls of abutments. Therefore, in this study, the coordinates of the center of each ball were depicted without using a best-fit algorithm, and the distance between the centers of the balls was calculated.

### Errors in trueness and precision

First, errors in the trueness and precision of the scanners in measuring the distance between the abutments in six pairs of abutments were investigated. In the intraoral scanner group, there was a significantly greater error in both trueness and precision in all regions than in the laboratory scanner group. Neither trueness nor precision significantly differed between the laboratory scanners in any region. Laboratory scanners capture an object from various angles over a wide area using a high-performance camera, with the object fixed at a certain distance. Consequently, multiple pieces of captured data are automatically combined into the STL format by the applied software. Because a dentist’s skill in preparing impressions does not affect the data quality, laboratory scanners are highly accurate with a very small deviation in trueness and precision. Intraoral scanners have a smaller camera than laboratory scanners and capture data over a small area per scan. By combining image data from each scan as needed, 3D images are combined like beads into the STL format. For this reason, image distortions from each data synthesis process are accumulated (Figs. 8, 9), resulting in greater errors [31]. In some regions, TDS, which uses scan powder to prevent reflection, produced a significantly smaller error than TR3, which requires no powder, as specified by its scan protocol. Our preliminary experiment showed greater accuracy on using TR3 with powder than on using TR3 without powder. In this study, TR3 was used without powder, according to the scan protocol. A comparison between the intraoral scanners showed that powder-free TR3 produced greater errors in trueness and precision than powder-requiring TDS at inflection points, such as that within the distance between A and D. A previous study reported that software often fails to capture simple contours, such as incisal edges, at inflection points, accurately [34]. In measuring the distance between A and C, powder-free TR3 produced a greater precision error than powder-requiring TDS, suggesting that scanners used with powder have greater precision. However, this study measured the distance between metal ball abutments. The finding that powder-free TR3 produces greater errors than powder-requiring TDS is consistent with those of previous studies [30]. This finding indicates the importance of powder for the prevention of reflection in terms of accuracy, suggesting that powder appears to be useful in patients with many metal restorations.

Meer et al. [32] reported that the scanning principles used for intraoral scanners cause errors in accuracy. The scanning principles of the scanners tested in our study can be classified into two main types: active wavefront sampling (AWS) for TDS and the confocal method for TR3. Regardless of the scanning principle, the intraoral environment, including improper scanning distance, patient body movements, and saliva secretion, can affect the accuracy of intraoral scanners [32]. The accuracy of scanning using intraoral scanners has been reported to be dependent on the practitioner’s skill [35]. This appears to be based on procedural factors, such as scanner manipulation skill during scanning, distance from the objective, and hand jiggling. However, it is difficult to eliminate these effects in routine practice. Dentists need sufficient training on the use of intraoral scanners and accurate operational procedures before the application of the technique in routine practice [35].

In actual clinical settings, impressions of various sizes of dental arches are taken for single-to-multiple-tooth restorations. In particular, taking impressions for long-span restorations involving the left and right teeth is not easy. This study investigated the difference in accuracy of intraoral scanners by measuring the distance between the ball abutments in six pairs of ball abutments, which simulated cases of multiple implants. The error in measurements with the intraoral scanners tended to increase with the distance between the ball abutments. Therefore, intraoral scanners are not suitable in terms of accuracy for capturing optical impressions of full arches, and this trend is consistent with that in previous studies [36]. Therefore, optical impression techniques can be used for dental implant treatment using a verification index.

Laboratory scanners require a conventional silicone rubber impression material and plaster cast. An in vitro study showed that the errors in the fabrication of a model were 20.4 ± 2.2 µm in trueness and 12.5 ± 2.5 µm in precision [37]. Although the aforementioned study demonstrated the high trueness and precision of laboratory scanners, it did not account for the shortcomings of these scanners, such as errors resulting from the deformation of impression materials or expansion of plaster, which dentists encounter in actual clinical settings. Although the intraoral scanners produced greater errors than the laboratory scanners, the optical impression technique was still superior to the conventional method, because impression material-related errors can be avoided. Moreover, hardware and software improvements are expected to improve the accuracy of intraoral scanners. Our results indicate that intraoral scanners can be used in patients with two to three missing teeth. This is because the accuracy of the intraoral scanners in regions with two or three missing teeth was comparable to that in a previous report [37–39]. The use of intraoral scanners in actual oral environments needs to be evaluated in vivo in future research. Furthermore, our results demonstrated that error values lower than 60 µm were observed on scanning regions with two or three missing teeth using intraoral scanners. Because the cement space should be 60–100 µm, these error values must account for this space. These findings suggest that intraoral scanners can be used to treat patients with a few missing teeth. On the other hand, screw retain system is often employed and may require higher accuracy than cement retain system. However, our study only demonstrated the accuracy of optical impression and further experiments should be required to clarify whether intraoral scanners can be applied to the screw retain system, or not.

## Conclusions

1. The laboratory scanners produced stable trueness and precision regardless of the distance between the abutments. However, in measurements with laboratory scanners, errors originating from impression materials and plaster models must be considered.
2. On using the intraoral scanners, the error values corresponding to trueness and precision suggested that these scanners are suitable for dental implant treatment in patients with a few missing teeth.
3. It is difficult to take impressions with intraoral scanners in the case of full-arch implant treatment. Combining the use of these scanners with conventional methods, such as a verification index and/or other conventional techniques, may be required.
4. Scan powder might improve the accuracy in the case of multiple missing teeth or edentulous jaw.

## Data Availability

Not applicable.
